# The association between cigarette smoking attitudes and social capital among Iranian health and medical students: a cross-sectional study

**DOI:** 10.1186/s12889-021-11435-y

**Published:** 2021-07-10

**Authors:** Hamideh Zahedi, Mohammad Hasan Sahebihagh, Parvin Sarbakhsh, Leila Gholizadeh

**Affiliations:** 1 Student Research Committee, Nursing & Midwifery Faculty, Tabriz University of Medical sciences, Tabriz, Iran; 2 Tabriz Health Service Management Research Center and Department of Community Health Nursing, Tabriz University of Medical Sciences, Tabriz, Iran; 3 Department of Statistics and Epidemiology, School of Public Health, Tabriz University of Medical Sciences, Tabriz, Iran; 4 Faculty of Health, University of Technology Sydney, Sydney, Australia

**Keywords:** Attitude, Cigarette smoking, Social capital, University students

## Abstract

**Background:**

Smoking remains a leading public health challenge globally. As a psychosocial determinant of health, social capital can influence health attitudes and behaviors, and thus it may have the capacity to reduce smoking rates. The aim of this research was to examine the association between social capital and attitudes towards smoking among university students.

**Methods:**

This cross-sectional study was conducted among 538 health and medical students, recruited using the proportionate sampling method. Participants’ social capital and attitudes toward smoking were assessed using the social capital questionnaire (SCQ) and the scale of cigarette smoking attitude (CSA). Data were analyzed using descriptive statistics, Pearson correlation coefficient, and the multiple regression analysis.

**Results:**

About one in four health and medical students reported smoking, either currently or in the past, and 30% had either positive or indifferent attitudes towards smoking. The mean scores of the SCQ and the CSA were 105.1 ± 19.7 and 48.6 ± 11.2, respectively. There was a statistically significant negative association between the SCQ and the CSA scores (r = − 0.24; *p* < 0.001). In the regression analysis, the SCQ scores were also negatively and statistically significantly associated with the CSA scores, after controlling potential confounders (B: -0.09; 95% CI: − 0.13 to − 0.004).

**Conclusions:**

As future healthcare providers, who are expected to take the primary role in reducing smoking rates in the community, health and medical students should be supported to develop appropriate attitudes towards smoking. Promoting positive social capital among university students has the capacity to improve their attitudes towards smoking. Possessing negative attitudes towards smoking should hopefully reduce smoking behaviors among future health professionals and improve their participation in anti-smoking campaigns.

## Introduction

Cigarette smoking is one of the most significant public health concerns worldwide, accounting for more than 5 million deaths annually, with the figure expecting to grow [1]. According to a World Health Organization projection, more than 8 million deaths per year will occur due to tobacco smoking by 2030 [2]. More than 80% of these deaths are anticipated to occur in low- to middle-income countries [3]. Smoking incareses the risk developing cancer, heart disease, stroke, and chronic lung disease [2]. Somkers are also more likely to pyschological problems, such as anxiety and depression [4].

The prevalence of cigarette smoking is high among university students globally, including students who study in health-related fields [5–8]. A recent study in Lithuania showed that 41.1 and 55.7% of local and international dental male students were smoking, respectively [6]. An international study which compared smoking rates in among medical students reported higher smoking rates among medical students in Spain and Turkey, but lower rates among Australia and the United States students [9]. In Iran, cigarette smoking rates among medical students vary between 15.2–23.8% [10, 11]. As the future role models, the high rates of smoking among health and medical students are concerning [1], particularly that health professionals are expected to play a crucial role in anti-smoking campaigns [12].

Many factors affect health-related behaviors [13–16], including social contexts which reinforce desirable behaviors [13–16]. Social capital is defined as the characteristics of social structures, such as levels of interpersonal trust, reciprocal norms, and mutual assistance, that create resources for individuals and facilitate collective actions [1]. Social capital is considered a significant psychosocial determinant of health [17], influencing various health behaviors and outcomes [18], such as violence and physical and psychological health [19]. Social capital affects health through several mechanisms: norms and attitudes that influence health behaviors, psychosocial networks that increase access to health care, and psychosocial mechanisms that increase self-esteem and influence health behaviors through interpersonal relationships [20]. Positive social capital is linked with lower social harms, substance use, smoking, alcohol consumption [21, 22], and improved determinants of health, such as education [17].

However, it should be noted that social capital itself is not a protective factor against risk behaviors, such as smoking. While healthy social capital can positively affect health attitudes and behaviors by reinforcing positive health messages, unhealthy social capital can impose negative effects. For example, Albert-Lőrincz et al. (2020) found that participation in neighborhood communities and social programs increased the risk of smoking among the youth in Romania [13]. Similarly, a study including participants from Flemish Belgium, Canada, Romania, and England, reported that social capital related to friends increased the likelihood of smoking among adolescents [16]. Due to peer pressure, unhealthy social capital can increase the chance of smoking [14], while healthy social capital strengthens cooperation and relations of mutual support in the communities and nations. Therefore, it can be a valuable tool in combating unhealthy behaviors, such as crimes and substance use [21, 22].

Several studies have examined the link between social capital and smoking behaviors, and the results of these studies have consistently supported the important role of social capital in influencing smoking behaviors [1, 13, 22, 23]. However, little is known about the impact of social capital on shaping attitudes towards smoking among health and medical students in Iran and internationally. This study aimed to examine the role of social capital in shaping attitudes toward smoking among Iranian health and medical students. This is an important topci to explore, considering the high rates of smoking amongst Iranian university students and some other nations and given that health and medical students are the critical members of future health care systems.

## Methods

### Study design and sampling

This cross-sectional study was conducted at Tabriz University of Medical Sciences, Tabriz, Iran, from September to December 2019. The university has been dedicated to educating students for more than 73 years and serving the community by delivering public health and clinical services. Approximately 8944 students were enrolled in a broad range of health and medical courses in the year 2020. The study population comprised the university students who were enrolled in a health and medical course for the academic calendar of 2019–2020, either in undergraduate or postgraduate levels. Students who were in clinical placements, those who did not provide informed consent to the study, or filled out the questionnaires incompletely were excluded.

The correlation sample size formula (N = [(Z_α_ + Z_β_)/C]^2^ + 3, C = 0.5 * ln[(1 + r)/(1-r)]) was used to determine the sample size that would allow examination of the association between cigarette smoking attitude and social capital considering the value of *p* < 0.05, power equal to 0.80, and the tendency to discover a correlation (r) equal to or greater than − 0.12 between the study variables [24]. Based on sample size formula, a total of 543 participants were required. One or two classes from each faculty were chosen randomly using the proportionate sampling method. All students in the selected classes (*n* = 580) were invited to participate in the study, of whom 24 students rejected the invitation, and 556 students completed the study questionnaires. Eighteen questionnaires were incomplete and excluded, leaving 538 questionnaires for final analysis (response rate: 92.8%).

### Data collection

Data were collected using three questionnaires; a researcher-developed questionnaire was used to collect data on demographic characteristics, such as age, sex, marital status, faculty of study, year of study, and history of cigarette smoking. Participants’ attitudes towards smoking were assessed using the scale of cigarette smoking attitude (CSA) [25]. This self-administered questionnaire is composed of 32 items and three cognitive (9 items), emotional (11 items), and behavioral (12 items) dimensions. The cognitive dimension addresses personal opinions and beliefs about an object or a thought; the emotional dimension addresses emotional feelings linked to beliefs, and the behavioral dimension measures readiness to respond in a certain way. The response options are ordered from one (disagree) to three (agree), and the total CSA scores can range between 32 and 96, with higher scores representing more positive attitudes towards smoking [25]. The total scores are grouped into three categories of negative attitude (32–53), indifferent attitude (54–74), and positive attitude (75–96) [25]. The face validity and internal reliability of the original CSA were good, with Cronbach’s alpha coefficient for the whole scale and the cognitive, emotional, and behavioral dimensions being repotred as 0.87, 0.74, 0.82, and 0.87, respectively [25]. The corresponding values of 0.91, 0.75, 0.88, and 0.90 were obtained in our study.

Social capital was measured using the Social Capital Questionnaire (SCQ), developed by Onyx and Bullen in 2000 [26]. The questionnaire consists of 34 items mesauring eight dimensions of participation in the local community (7 items), social agency/proactivity in a social context (7 items), feelings of trust and safety (5 items), neighborhood connections (5 items), family and friends connections (3 items), tolerance of diversity (2 items), the value of life (2 items), and work connections (3 items). Each item is provided with a 5-point Likert-type response scale ranging from 1 to 5 (1 = not at all, 2 = rarley, 3 = sometimes, 4 = often, and 5 = always). The total SCQ scores can range from 34 to 170, with higher scores indicating higher social capital [26]. Yari et al. (2014) translated the questionnaire into Persian and examined the construct validity and internal reliability of the Persian SCQ on a sample of medical students, with the reported Cronbach’s alpha of 0.79. They suggested the following categories for the total scores: good/high social capital (133–180), moderate social capital (85–132), and poor/low social capital (36–84) [27].

### Data analysis

Data were coded and analyzed by IBM SPSS Statistics version 22. Descriptive statistics, including frequency, percentage, mean, and standard deviation, were used to summarize the students’ demographic characteristics, the CSA, and the SCQ scores. The Chi-Square analysis was used to compare the CSA and the SCQ scores according to the participants’ demographics. Social capital was considered as an independent variable and cigarette smoking attitude as the dependent variable. The association between social capital and cigarette smoking attitudes was assessed using multiple linear regression analysis, adjusting for potential confounders. Variables with *p* < 0.1 were entered into the regression analysis [28]. Pearson correlation analysis was applied to test the association between social capital and cigarette smoking attitudes. The level of statistical significance was set at *p* < 0.05.

## Results

Out of 538 participants, 301 (59.9%) participants were female and 56 (10.4%) married. Most participants (68.7%) lived with their families, and 131 (24.3%) students reported smoking cigarettes either in the past or currently. The distribution of the CSA scores according to the demographic characteristics of participants is shown in Table 1. The results of Chi-Square analyses showed that there was a statistically significant association between the CSA scores and sex (*p* < 0.001), year of study (*p* = 0.021), and smoking cigarettes either in the past or currently (*p* < 0.001).

**Table 1:** The distribution of the CSA scores according to the demographic profile (*n* = 538)

Variables N(%)	Positive	Indifferent	Negative	***p***-value
Sex				
*Male*	11 (4.6)	83 (35.0)	143 (60.4)	
*Female*	1 (0.4)	66 (21.9)	234 (77.7)	< 0.001*
Age (years)				
≤ *21*	4 (1.6)	62 (24.2)	190 (74.2)	0.116
*> 21*	8 (2.8)	87 (30.9)	187 (66.3)	
Marital status				
*Single*	10 (2.1)	135 (28.0)	337 (69.9)	0.710
*Married*	2 (3.6)	14 (25.0)	40 (71.4)	
Residence				
*With family*	9 (2.4)	107 (28.9)	254 (68.7)	0.549
*Student dormitory*	3 (1.8)	42 (25.0)	123 (73.2)	
Year of study				
*Year one*	1 (0.9)	21 (18.8)	90 (80.3)	
*Year two*	8 (3.2)	82 (32.0)	166 (64.8)	0.021*
*Year three*	1 (1.2)	17 (19.7)	68 (79.1)	
*Year four& above*	2 (2.4)	29 (34.5)	53 (63.1)	
Experience of cigarette smoking				
*Yes*	10 (7.7)	78 (59.5)	43 (32.8)	< 0.001*
*No*	2 (0.5)	71 (17.4)	334 (82.1)	
Field of study				
*Medicine*, *Dentist*, *Pharmacy*	5 (1.4)	104 (29.2)	247 (69.4)	
*Nursing or Midwifery*, *Health sciences or Nutrition*	7 (3.8)	45 (24.7)	130 (71.5)	< 0.125

* Significant at *p* < 0.05

The total scores of the CSA, the SCQ, and their dimensions are presented in Table 2. The mean total scores of the CSA and the SCQ were 48.6 ± 11.2 and 105.1 ± 19.7, respectively.

**Table 2:** The mean scores of cigarette smoking attitudes, its dimensions, and social capital

Variables	Mean ± SD^a^
**Total CSA**	**48.6 ± 11.2**
*Cognitive*	13.2 ± 3.3
*Emotional*	17.1 ± 4.7
*Behavioral*	18.1 ± 5.6
**Total SCQ**	**105.1 ± 19.7**
*Participation in the local community*	16.1 ± 5.9
*The social agency, or proactivity in a social Context*	22.4 ± 4.5
*Feelings of trust and safety*	14.3 ± 3.4
*Neighborhood connections*	13.3 ± 3.7
*Family and friends connections*	10.4 ± 2.7
*Tolerance of diversity*	5.7 ± 1.9
*Value of life*	6.3 ± 1.9
*Work connections*	9.5 ± 2.5

^a^ Standard deviation

The distribution of different levels of the CSA and the SCQ is shown in Table 3. About 30% of the participants had either positive or indifferent attitudes towards smoking. The majority of participants (76.6%) had a moderate level of social capital.

**Table 3:** The distribution of different levels of the CSA and the SCQ

Variables	N(%)
**The CSA**	
*Positive (75–96)*	12 (2.2)
*Indifferent (54–74)*	147 (27.4)
*Negative (32–53)*	379 (70.4)
**The SCQ**	
*Good/High (133–180)*	48 (8.9)
*Moderate (85–132)*	409 (76.0)
*Poor/low (36–84)*	81 (15.1)

Pearson’s correlation coefficients revealed a statistically significant negative correlation between the SCQ and CSA scores (r = − 0.24, *p* < 0.001). The results of the multiple linear regression analysis are shown in Table 4. The model indicated that social capital scores were negatively associated with cigarette smoking attitude scores (B = − 0.09, 95% CI: − 0.13 to − 0.04) after adjusting for potential confounding variables. The CSA scores were statistically significantly associated with gender, with male students showing more positive attitudes towards smoking than female students (B = 0.12, 95% CI: 0.06 to 0.17). The year of study was also statistically significantly associated with the CSA scores; in that year one students showed more negative attitudes towards smoking than students in year four and above (B = − 0.14, 95% CI: − 0.24 to − 0.04). Also, students who had smoking experience (current or past) presented more positive attitudes towards smoking than those without such experience (B = 0.35, 95% CI: 0.28 to 0.41).

**Table 4:** Multiple linear regression of the association between cigarette smoking attitudes and social capital

Variables	B	SE	95% CI	***p***-value
Age (reference: 22 and above)	0.24	0.03	[−0.04 to 0.09]	0.450
Sex (reference: Female*)*	0.12	0.03	[0.06 to 0.17]	< 0.001^a^
Year of study (reference: Year four& above)				
*Year one*	− 0.14	0.05	[− 0.24 to − 0.04]	0.007^a^
*Year two*	0.02	0.04	[−0.06 to 0.10]	0.694
*Year three*	−0.07	0.05	[−0.16 to 0.02]	0.128
Experience of cigarette smoking (reference: No)	0.35	0.03	[0.28 to 0.41]	< 0.001^a^
Social capital	−0.09	0.02	[−0.13 to − 0.04]	< 0.001^a^

R2 = 0.292; Adjusted R2 = 0.282; F = 31.19; ^a^ Statistically significant association

## Discussion

This study aimed to examine the role of social capital in shaping health and medical students’ attitudes towards smoking. Although most students (70.4%) held negative attitudes towards smoking, the attitudes of 29.6% of the health and medical students were either positive or indifferent. The association between social capital and cigarette smoking attitudes was negative, meaning that students with more robust social capital held less positive attitudes towards cigarette smoking. About one in four students (24.3%) reported smoking either in the past or currently. This result is in line with previous studies reporting the smoking rate among Iranian medical students between 15.2 and 23.8% [10, 11]. These findings warrant closer attention. Health professionals are expected to take the primary role in educating society about the health risk of smoking. Even a small number of smoking physicians, who are seen as role models in society, can negatively impact on smoking control programs. Consequently, smoking prevention must be an essential part of curriculum requirements for all health-related disciplines [6] to help future health professionals gain a comprehensive understanding of the health risks of smoking and prepare them to take an active role in anti-smoking campaigns.

The available evidence suggests that university students generally held positive attitudes towards prevention and cessation of smoking [29]. For example, the majority of Indian medical students in a study believed that smoking should be banned in the society [12]. Similarly, a study among Beirut University students in Lebanon showed that students overall held favorable attitudes towards smoking bans and non-smoking policies in the public arenas [30]. Nevertheless, the high rate of positive or indifferent attitudes towards smoking among the health and medical students in our study is of concern. Similarly, Penhai et al. (2016) reported that the knowledge and attitdues of Irainain health and medical students about smoking was at a moderate level [31]. Further, Heydari et al. (2013) found that 23.1% of male university students, lecturers, and clergymen in Tehran had favorable attitudes towards smoking [32].

In the current study, a low level of social capital, male sex, being a senior student, and having smoking experience were associated with more positive attitudes towards smoking. The association between social capital and cigarette smoking attitudes was negative. This finding is in line with other similar studies conducted in Iran and internationally [1, 13, 22, 23, 33]. The results of a study on teenagers in Iran showed that social capital had a protective role against harmful health-related behaviors, such as smoking [33]. Similar results have been reported from studies conducted on different populations. For example, social capital was inversely associated with smoking behaviors among Chinese male employees [1]. Two studies conducted in the United States found that neighborhood cohesion, which is a dimension of social capital, was related to lower smoking behaviors among Asian American men [23] and Mexican Americans [22].

Further, Giordano and Lindstrom (2011) reported that trust and social participation, another dimension of social capital, was positively associated with smoking cessation. The study also found that the onset of smoking was higher in those who less actively participated in social activities [34]. In addition, a low social capital has been linked to increased risk of substance use, such as opium, cannabis, water pipe, alcohol, and oral tobacco in adolescents [35]. Yet, several studies have reported conflicting results. For example, the results of a study in Romania showed that the level of community engagement was not a protective factor against smoking [13]. Other studies found that the risk of smoking increased with larger networks of friends [36], and a higher level of social capital [16, 37]. These results suggest that the concept of social capital is not always positive [38].

Overall, the students in our study showed a moderate level of social capital. This finding is consistent with a previous similar study conducted in Tehran, Iran, reporting the health and medical sciences students’ social capital at a low-moderate level [27]. Nevertheless, studies that have assessed social capital among adolescents in general, both in Iran and outside, reported a strong social capital among adolescents [21, 39]. Compared to adolescents in general, the relatively lower level of social capital among health and medical students may indicate that university students have limited opportunities to develop social capital likely due to their university commitments. Further, most students in the current study were still living with their families, which can affect development of certain aspects of social capital. This is supported by the finding that the highest level of social capital among the participants was in the dimension of the family and friends connections and lowest in the dimension of participation in the local community. Similar findings were found by Moghaddam et al. (2016), who examined social capital among students at Jahrom University of Medical Sciences, Iran [40]. These results suggest that families and authorities should provide a sound basis for the growth of social capital, among adolescents and youth, particularly in the dimention of commuinity engagmnet, by creating opportunities for social interactions and participation.

Female sex was associated with more negative attitudes towards smoking, which is an expected finding. Generally, smoking rates are higher among men than women [41], and men hold less negative attitudes towards smoking than their female counterparts [42–44]. Gender differences in smoking are, to a great extend, related to socio-cultural differences [7]. Generally, smoking is more accepted culturally for men than women. It is also known that family relationships have a significant impact on smoking behaviours, and women are more likely than men to be affected by family factors [14]. A study found that American adolescent girls are more likely than boys to smoke during family conflicts [45]. In Iran, smoking is more accepted for men socially and culturally, while it is viewed as an abnormal behaviour for women. This can explain the lower rate of smoking and more negative attitudes towards this behaviour among the female students in our country.

First-year students held more negative attitudes towards smoking than those in year four and higher. This finding is in line with the past research, suggesting that the pervasiveness of smoking increases significantly according to years of study [46, 47]. The rate of smoking is generally higher in senior university students than in freshman stduents [48], and it is well known that people who smoke hold more positive attitudes towards smoking than those who do not smoke [44, 49, 50]. Our study further confirmed this by revealing a significant association between smoking experience (either current or past) and attitudes towards smoking, in that students who were either current or past smokers were more likely to possess positive attitudes towards smoking.

### Limitation of the study

To our knowledge, this study is the first to investigate the association between social capital and attitudes towards smoking among health and medical students in Iran. There are some limitations to consider in interpreting the results. First, a cross-sectional study design is weak in establishing causal relationships, although using the multiple regression analysis helped the researchers control for the effects of some potential confounders. It is suggested that future studies use other research designs, such as longitudinal methodology or interventions, to confirm the findings. Second, the data were collected using a self-report questionnaire. In all self-report questionnaires, social desirability can affect the accuracy of provided responses, particularly that the participants in this study were completing a degree in a health-related discipline. To reduce this type of bias, the participants were provided with privacy to complete the questionnaires and assured about the anonymity of the survey. Finally, our study was performed among students at Tabriz University of Medical Sciences; hence, caution should be exercised when generalizing the results to other universities in Iran and ouside.

## Conclusions

This study found that nearly one in four health and medical students were current or past smokers, and about 30% of the students held either positive or indifferent attitudes towards smoking. As future healthcare providers who are expected to play a crucial role in anti-smoking programs, these findings are concerning, and suggest that universities should implement innoviative evdienced based effective methods in traninign of medical and health stduents about the health risk of smoking. Social capital among the study participant was at a moderate level, and it was negativly associated with the students’ attitudes towards smoking. As a factor that can improve negative attitudes towards smoking, families and university authorities should promote social capital amongst youth through providing social interactions and participation opportunities to help reduce smoking rates in the society.

## Data Availability

The datasets used and/or analysed during the current study are available from the corresponding author on reasonable request.

## Abbreviations

CSA: Cigarette smoking attitude
SCQ: Social capital questionnaire
